# Exposure to gestational diabetes mellitus increases subclinical inflammation mediated in part by obesity

**DOI:** 10.1093/cei/uxae010

**Published:** 2024-02-09

**Authors:** Andrea Musumeci, Colm John McElwain, Samprikta Manna, Fergus McCarthy, Cathal McCarthy

**Affiliations:** Department of Pharmacology and Therapeutics, Western Gateway Building, University College Cork, Cork, Ireland; Department of Pharmacology and Therapeutics, Western Gateway Building, University College Cork, Cork, Ireland; Department of Obstetrics and Gynaecology, Cork University Maternity Hospital, Cork, Ireland; Department of Obstetrics and Gynaecology, Cork University Maternity Hospital, Cork, Ireland; Department of Pharmacology and Therapeutics, Western Gateway Building, University College Cork, Cork, Ireland

**Keywords:** gestational diabetes, obesity, immune cell activation, cytokines, adipose tissue, placenta

## Abstract

Gestational diabetes mellitus (GDM) is a frequent and serious complication of pregnancy, often associated with obesity. Metabolic dysfunction and metainflammation are evident in both obesity and GDM. In this cross-sectional study, we aimed at defining the direct contribution of the immune system in GDM, across the main metabolic tissues, specifically focussing on elucidating the roles of obesity and GDM to the clinical outcome. Using immunoassays and multicolour flow cytometry, cytokine profiles and immune cell frequencies were measured in maternal circulation and central metabolic tissues [placenta and visceral adipose tissue (VAT)] in GDM-diagnosed (*n* = 28) and normal glucose tolerant (*n* = 32) women undergoing caesarean section. Participants were sub-grouped as non-obese [body mass index (BMI) < 30 kg/m^2^] or obese (BMI ≥ 30 kg/m^2^). Unsupervised data analysis was performed on the flow cytometry data set to identify functional alterations. GDM obese participants had significantly elevated circulating IL-6 and IL-17A levels. GDM non-obese participants had elevated circulating IL-12p70, elevated placental IL-17A, and VAT IFN-γ production. Unsupervised clustering of immune populations across the three biological sites simultaneously, identified different NK- and T-cell phenotypes that were altered in NGT obese and GDM non-obese participants, while a classical tissue monocyte cluster was increased in GDM obese participants. In this study, there was significant evidence of subclinical inflammation, and significant alterations in clusters of NK cells, T cells, and tissue monocyte populations in GDM. While increased adiposity assimilates with increased inflammation in the non-pregnant state, this overt relationship may not be as evident during pregnancy and warrants further examination in future longitudinal studies.

## Teaser

Gestational diabetes mellitus (GDM) is a frequent and serious complication of pregnancy, often associated with obesity. In this study, there was significant evidence of subclinical inflammation in maternal circulation and in both placental and visceral adipose tissue. However, no direct causal role could be attributed to any specific immune cell population in GDM.

## Graphical Abstract

**Graphical Abstract:**
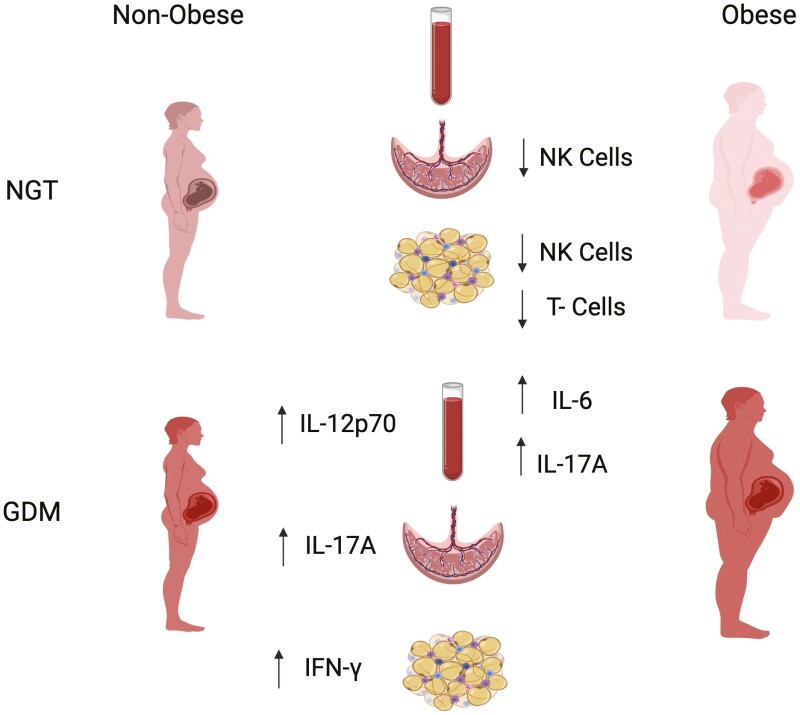

## Introduction

Gestational diabetes mellitus (GDM) is a serious obstetric complication that affects approximately 10–15% of pregnancies [1, 2]. It is characterized by hyperglycaemia due to an insufficient insulin response to compensate for the insulin-resistant state of pregnancy, a well-defined physiological response necessary to provide sufficient glucose for maternal and foetal requirements [3]. The global prevalence of obesity in pregnancy is rapidly increasing, resulting in an elevated risk of cardiometabolic complications in both mother and baby. Obesity is an established risk factor for GDM, and maternal body mass index (BMI) is a major contributor to GDM diagnosis with a 6.79-fold increased risk in obese women and 2.29-fold increased risk in overweight women [4].

Maternal obesity is associated with chronic, low-grade inflammation termed ‘metainflammation’ or ‘metabolically induced inflammation’, as opposed to an acute inflammatory response [5, 6]. Metainflammation is a hallmark of obesity in the non-pregnant state and is proposed to be orchestrated by metabolites and lipids. During GDM pregnancy, metainflammation is thought to develop in metabolic organs including the pancreas, visceral adipose tissue (VAT), and the placenta [5, 7, 8], resulting in disruption of metabolic homeostasis which potentially precipitates insulin resistance and hyperglycaemia. This immune dysregulation encompasses several cell types across both innate and adaptive immunity [9].

The placenta is the central metabolic organ during pregnancy, acting as a conduit between the foetal and maternal circulations. The placenta provides necessary nutrients to the developing baby, the delivery of which is regulated by a complex interaction including insulin signalling, cytokine mediators, and insulin responsiveness. Maternal adipokines and metabolic hormones also have a direct impact on placental function by modulating placental nutrient transport [10]. Obesity-related disruptions, therefore, have a major impact on placental structure and function [11]. By directly influencing placental function, maternal obesity and GDM may also indirectly program the foetus for later cardiometabolic disease.

Adipose tissue (AT) equally plays an important role during pregnancy, regulating systemic glucose homeostasis by efficiently storing lipids and modulating adipokine secretion. This maintains metabolic homeostasis and prevents glucotoxicity and lipotoxic-mediated effects. Adipocyte dysfunction is a prominent driver of insulin resistance, which is mediated by several molecular mechanisms, including the excessive release of monocyte chemoattractant protein 1 (MCP-1) with subsequent macrophage infiltration and metainflammation [12]. The pro-inflammatory status of AT is a well-established feature of obesity, with several tissue infiltrating immune cells involved [13]. Compared to subcutaneous AT, VAT shows a higher degree of inflammation in obesity and is associated with an increased risk of type 2 diabetes (T2D), hypertension, and dyslipidaemia [14].

While soluble immune factors play a central role in tissue communication, increasing importance has been identified for extracellular vesicles (EVs) [15, 16]. EVs are cell-derived particles enclosed in a lipid bilayer and are released by all cellular organisms. They comprise exosomes and larger vesicles and play important roles in basic processes of innate and adaptive immunity [17]. In a recent murine model, infusion of EVs isolated from human GDM pregnancies induced glucose intolerance in otherwise healthy mice [18], suggesting a direct role of EVs in the modulation of the disease.

In this study, we set out to define the immune cell and soluble mediator contributions across the maternal circulation and in both placental and VATs in GDM, with an emphasis on delineating those perturbations which may be specific to GDM pathology and/or obesity during pregnancy.

## Methods

### Study design and participant recruitment

GDM-diagnosed (*n* = 28) and healthy normal glucose tolerant (NGT) control (*n* = 32) participants were recruited from Cork University Maternity Hospital (CUMH), Ireland between 2019 and 2022 as part of the COMRADES study, a non-interventional cohort study of nulliparous singleton pregnancies with the aim of characterizing metainflammation in GDM. Inclusion criteria for all study participants included women aged between 18 and 50 years, nulliparous pregnancy, singleton pregnancy as determined by ultrasound scan, opted for an elective caesarean section, and had provided written informed consent. As per national HSE guidelines, selective screening for GDM was carried out on identified at-risk pregnant women between 24 and 28 weeks gestation. GDM was diagnosed where fasting blood glucose levels >5.1 mmol/L.

Healthy NGT control participants were defined as pregnant women who had not been diagnosed with GDM, as per the guidelines mentioned earlier, and did not present with any other diagnosed comorbidities. Exclusion criteria included women who had twin or multiple pregnancy, were multiparous, had already started to labour, were outside the age range of 18–50 years of age, or had an emergency caesarean section. Women with known pregnancy or other health-related conditions—namely pre-pregnancy essential hypertension; known liver or renal diseases; known human immunodeficiency viruses, hepatitis B, or hepatitis C positive; known systemic lupus erythematous; known antiphospholipid syndrome; and known or suspected foetal anomalies—were excluded from the study.

Although our study cohort size for each group was approximately *n* = 30, smaller numbers were used for individual assays where technical and/or experimental limitations were present. Where possible, samples were chosen from the pool of samples to allow for matching by maternal age and/or BMI. Where this occurs, it is explicitly stated. Where this was not possible for larger sample numbers, samples were chosen to exclude those participants with either extreme age and/or BMI parameters and to include those participants for which complete biological sample collection was available (fasting blood, placental tissue, and VAT).

### Sample collection

Fasting blood samples were collected from study participants pre-surgery on the day of elective caesarean section. Blood samples were collected in ethylenediaminetetraacetic acid (EDTA) vacutainer tubes for plasma collection and vacutainer serum tubes for serum collection. Samples were centrifuged at 2400 × *g* for 10 minutes at 4°C before plasma and serum were collected. Samples were stored at −80°C until analysis was performed.

VAT was collected from one site during caesarean section and processed within 30 minutes post-surgery. Placental tissue samples were obtained immediately post-caesarean section. Placental tissue was sectioned following removal of decidua from 5 regions of each placenta within 30 minutes post-surgery. Additionally, tissue samples were taken for flow cytometry and explant culture experiments.

### Peripheral blood mononuclear cell isolation

Following sample collection, 1–3 ml of whole blood collected in EDTA vacutainer was overlaid onto 5 ml Histopaque density gradient (3.1 ml Histopaque 1119 overlaid by 1.9 ml Histopaque 1077) in a 15-ml falcon tube followed by centrifugation at 700 × *g* for 30 minutes, with reduced acceleration and deceleration. Cells at the interface were collected with a pasteur pipette and washed in 5 ml PBS. This suspension was filtered through a 70 µm cell strainer (Sigma CLS431751) to remove cells aggregates. The resultant cell suspension was pelleted by centrifugation at 300 × *g* for 5 minutes. Supernatant was discarded and the cell pellet was stained for flow cytometry analysis.

### Isolation of tissue infiltrating leukocytes

Placental tissue (15–20 g) and omental VAT (5–10 g) were stored in Tissue Preservative Buffer (154 mM NaCl, 1.6 mM CaCl_2_, 10 mM HEPES, 250 mM sucrose, 5.4 mM K_3_PO_4_, and 1.2 mM MgCl_2_ in sterile, distilled water) on ice until processing. Samples were processed within 2 hours from acquisition. Briefly, tissue pieces were rinsed twice in ice-cold, sterile PBS to remove blood, followed by mechanical dissociation using a scalpel to scrape the parenchyma and obtain a fine fragmentation, taking care to remove fibrous tissue and blood clots. The tissue was then digested in 20 ml of cell culture medium [DMEM/F-12 (Gibco 31330038), 10% FBS (heat-inactivated; Gibco 16140071), 1% penicillin–streptomycin (10 000 U/ml; Gibco 15140122)] containing 0.2% (w/v) collagenase type I (Sigma C0130) and 0.02% (w/v) DNAse I (Sigma D5025), at 37°C in a water bath for 1 hour (placenta) or 45 minutes (omental VAT), with occasional agitation to facilitate enzymatic digestion. Afterwards, the suspension was carefully decanted through sterile gauze (Steroswab 1905/SF) to remove undigested tissue fragments, and the tube and gauze rinsed with 20 ml of sterile PBS. For AT only, the resulting cell suspension was centrifuged for 5 minutes at 200 × *g*, and the layer of floating adipose droplets was carefully removed with a Pasteur pipet. Afterwards, both tissues’ cell suspension was pelleted by centrifugation at 300 × *g* for 5 minutes, and the supernatant was carefully removed with a Pasteur pipet. The resulting pellet was resuspended in 10 ml sterile PBS and overlaid onto 10 ml of Histopaque gradient (6 ml Histopaque 1119 overlaid with 4 ml Histopaque 1077), and mononuclear cells isolated as described earlier. Supernatant was discarded and the cell pellet was stained for flow cytometry analysis.

### Flow cytometry analysis

Isolated peripheral blood mononuclear cells, placental and omental immune cells were resuspended in FACS buffer and kept on ice throughout. Approximately 1 × 10^6^ cells of the single cell suspensions were placed in 5 ml round-bottomed tubes (Falcon 352008; one for each panel) and centrifuged at 300 × *g* for 5 minutes; supernatants were discarded. Cell pellets were incubated with 50 µl FC blocking buffer [10% human serum (heat-inactivated; Sigma S1-M) in 1× PBS (without Ca^2+^/Mg^2+^; Sigma P4417)] containing Fixable Viability Dye eFluor™ 455UV (eBioscience 65-0868-14; 1:1000) on ice for 10 minutes. Fifty microliters of each antibody mix were added and cells were incubated for 20 minutes on ice. Staining panels are reported in Supplementary Table ST1. Cell suspensions were then washed with 2 ml FACS buffer [EDTA 2 mM (Sigma E6758), FBS 2% (heat-inactivated; Gibco 16140071) in 1× PBS] and centrifuged at 300 × *g* for 5 minutes. Supernatant was discarded and 100 µl of fixation buffer [PFA 4% (Sigma 158127) in 1× PBS] was added. Cell suspensions were incubated for 30 minutes at room temperature in the dark. Cell suspensions were then washed in 2 ml PBS and centrifuged at 300 × *g* for 5 minutes and supernatants were discarded. Finally, stained cells were resuspended in 150 µl PBS and stored in the dark at 4°C until acquisition.

Samples were analysed within 24 hours on a BD LSR II flow cytometer. Rainbow Calibration particles (BioLegend 422903) were used to standardize instrument settings for longitudinal analysis. Data were analysed using FlowJo (v10.8.1). All panels were gated for doublet exclusion followed by size selection using forward vs side scatter, and dead cells were excluded using viability dye gating. Each panel was then gated according to the target cells (Supplementary Figs S1–S3).

### Omental VAT and placental explant culture

Tissue explants were prepared from omental VAT samples collected from both NGT and GDM participants. 100 mg ± 10 mg of omental tissue were cultured in 12-well plates, containing 2 ml of culture media containing Medium 199 1× (Gibco 31150022), FBS (heat-inactivated) 10% (Gibco 16140071), penicillin–streptomycin (10 000 U/ml) 1% (Gibco 15140122), amphotericin B 0.25 µg/ml (Gibco 15290018). Tissue explants were also prepared from placental samples collected from both NGT and GDM participants. Following the removal of outer membrane and connective tissue, 200 mg ± 20 mg of placental tissue (combined from three regions of the placenta) was cultured in 12-well plates, containing 2 ml of culture media. Placental explant media consisted of RPMI-1640 1× (Gibco 21875034), FBS (heat-inactivated) 10% (Gibco 16140071), penicillin–streptomycin (10 000 U/ml) 1% (Gibco 15140122), insulin 1 µg/ml (SAFC 91077C), hydrocortisone 0.1 µg/ml (Sigma H0888), retinol acetate 0.1 µg/ml (Sigma R0300000), and gentamicin 0.05 µg/ml (Sigma G1397). Tissue weight was recorded and used for normalization of all explant experimental analysis. Supernatants were subsequently stored at −80°C for further analysis.

### Extracellular vesicles

MACSPlex Exosome Kit, human (Miltenyi 130-108-813) was used according to the manufacturer’s instructions. Frozen plasma samples were thawed on ice, and 20 µl was used for the assay.

### Immunoassays

Immunoassays were used in the quantification of cytokines in either serum samples or placental and VAT explant supernatants from GDM and NGT participants. Multiplex panel of 13 cytokines including IL-1β, IFN-α2, IFN-γ, TNF-α, MCP-1, IL-6, IL-8, IL-10, IL-12p70, IL-17A, IL-18, IL-23, and IL-33 was measured using the LEGENDPlex Human Inflammation Panel 1 kit (BioLegend 740809), as per the manufacturer instructions.

### Statistical analysis

Statistical analysis of cytokine abundance was performed using GraphPad Prism (version 9.5.1). Significance was evaluated using linear regression, adjusting by maternal age, and followed by Benjamini–Hochberg correction for multiple testing. Statistical analysis of flow cytometry data was performed in R (version 4.2.2) using RStudio GUI (version 2023.03.0). For standard frequency analysis, data were exported from FlowJo as percentage of Live cells, and significance was evaluated using linear regression, adjusting by maternal age, and followed by Benjamini–Hochberg correction for multiple testing. For unsupervised clustering analysis, a maximum of 10 000 cells were exported in a new FCS file for each sample, gated on live cells, compensated values. The R package *CATALYST* (version 1.20.1) was used for analysis. Briefly, FCS files were imported for each panel independently, and each parameter transformed using logicle transformation (function *estimateLogicle*) and subsequently normalized across samples using Gaussian method (function *gaussNorm*). Non-redundancy scores were calculated and plotted, parameters with very low scores were excluded from further analysis. FlowSOM clustering and metaclustering were performed using the function *cluster*, with parameters *xdim* = *10*, *ydim* = *10*, and *maxK* = *20*. UMAP was calculated using the function *runDR*, with parameters *dr=“UMAP”* and *cells* = *NULL*. Significance was calculated using the function *diffcyt* (from the package *diffcyt*), with design based on the four experimental groups and contrasts set to test each group vs control (NGT_nonobese). Spearman’s rank correlation was calculated using the function *rcorr* from the package *Hmisc*, with parameter *type* = *“spearman”.* An adjusted *P*-value of <0.05 was accepted as statistically significant for all analyses.

## Results

A total of 60 participants were recruited for this study, including NGT (*n* = 32) and GDM-diagnosed pregnancies (*n* = 28). Clinical characteristics were recorded from electronic health records for each individual and any differences between participant groups were assessed. Participants’ characteristics are summarized in Table 1. Both NGT and GDM groups were further divided into non-obese and obese subgroups, based on BMI cut-off of 30. The NGT non-obese group is set as the control group for all analyses. There were no significant differences observed between study groups for maternal age, birth weight, placental weight, and foetal sex. Both NGT obese and GDM obese participants had a significantly higher BMI, while GDM obese participants had lower gestational age at caesarean delivery, relative to NGT non-obese participants.

**Table 1.** Clinical characteristics of the cohort used in the study

	Participant study group				ANOVA
	NGT		GDM		
	Non-obese (*n* = 19)	Obese (*n* = 13)	Non-obese (*n* = 10)	Obese (*n* = 18)	*P*-value
Maternal age (years)	33.16 ± 4.324	32.62 ± 5.059	35.30 ± 4.715	35.56 ± 5.903	0.2921
Maternal BMI (kg/m^2^)	23.63 ± 2.692	35.23 ± 5.833	25.80 ± 3.190	35.89 ± 3.628	<0.0001***
Gestational age (weeks)	38.92 ± 0.536	39.03 ± 0.409	38.59 ± 0.763	38.32 ± 0.623	0.0038**
Birth weight (g)	3446 ± 521.3	3532 ± 387.4	3295 ± 498.7	3564 ± 435.4	0.4969
Placenta weight (g)	656.1 ± 179.2	749.7 ± 110.8	670.7 ± 86.42	744.0 ± 164.9	0.2437
Foetal sex	M—6 F—13	M—7 F—6	M—5 F—5	M—9 F—9	0.5567
GDM intervention	N/A	N/A	Diet—80% (8) Metformin—10% (1) Insulin—10% (1)	Diet—22% (4) Metformin—39% (7) Insulin—39% (7)	

Data are presented as mean ± SD. One-way ANOVA followed by Dunnett’s multiple comparisons test was used to calculate the statistical significance of continuous variables. Chi-squared test was used for categorical variables. Significance levels: **P < 0.01, ***P < 0.001

### GDM participants show elevated levels of inflammatory cytokines in the maternal circulation

**Figure 1.**
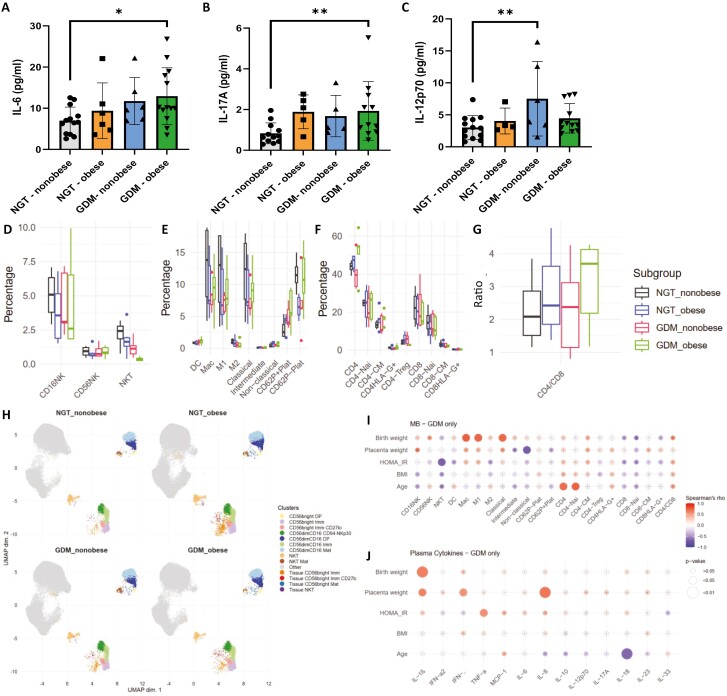
Pro-inflammatory cytokines and immune cell frequencies in maternal circulation. Abundance of inflammatory cytokines IL-6 (**A**), IL-17A (**B**), and IL-12p70 (**C**) measured in NGT (non-obese, *n* = 13; obese, *n* = 6) and GDM (non-obese, *n* = 6; obese, *n* = 13) pregnancies. (Frequency of NK-cell subsets (**D**), myeloid subsets (**E**), and T-cell subsets (**F**), shown as percentage of live leukocytes are measured in NGT (non-obese, *n* = 4; obese, *n* = 6) and GDM participants (non-obese, *n* = 5; obese, *n* = 5). (**G**) CD4/CD8 T-cell ratio. (**H**) UMAP was calculated using the full 12-colour panel for NK subsets. Correlations of clinical parameters with immune cell frequencies (**I**) and cytokine abundance (**J**). Statistical significance was calculated using linear regression with maternal age included as confounder, followed by Benjamini–Hochberg *P*-value correction for multiple testing. Differential analysis of cluster abundance was calculated using R (diffcyt package). The correlation was calculated using Spearman’s rank correlation. Significance levels: *P < 0.05, **P < 0.01, ***P < 0.001.

To define the cytokine profile in GDM, the levels of 13 cytokines in the maternal circulation at the time of delivery were determined. Circulating levels of pro-inflammatory cytokines IL-6 (*P* = 0.0125) and IL-17A (*P* = 0.009) were significantly higher in GDM obese patients compared to NGT non-obese, while circulating levels of IL-12p70 (*P* = 0.007) were significantly increased in GDM non-obese, compared to NGT (Fig. 1A–C). To understand which immune cell populations are responsible for these cytokine alterations, we analysed the frequency and activation status of several key innate and adaptive immune populations, by multicolour flow cytometry. There were no significant changes in the frequency or activation marker expression in any of the immune cell populations (Fig. 1D–G). Diagnosed treatment (diet-only, metformin, or insulin) had no significant effect on cytokines or immune cell frequencies or activation status in GDM participants.

Given the extensive coverage of our flow cytometry panel, we took advantage of unsupervised clustering algorithms (based on FlowSOM [19]) to assess how all markers behave across all cells simultaneously, allowing the identification of subsets or functional statuses that might otherwise be missed. However, there were no significant changes in the frequency of the detected clusters (Fig. 1H; Supplementary Fig. S4).

GDM is a predominantly metabolic pathology, with insulin resistance driving immune-metabolic alterations and affecting foetal phenotypes such as macrosomia [20]. To evaluate whether these alterations relate to plasma cytokine levels or immune cell frequencies, we measured their correlation with clinical parameters including homeostasis model assessment for insulin resistance (HOMA_IR), placental weight and birth weight, and confounders including maternal age and BMI, within the whole GDM population (Fig. 1I and J). There was a positive correlation of M1 macrophages and classical monocytes frequencies with birth weight (*ρ* = 0.733, *P* = 0.016 and *ρ* = 0.723, *P* = 0.018, respectively), while non-classical monocytes correlate inversely with placental weight (*ρ* = −0.714, *P* = 0.047). Pro-inflammatory cytokines IL-1β, IFN-γ, and IL-8 positively correlate with placental weight (*ρ* = 0.621, *P* = 0.018, *ρ* = 0.556, *P* = 0.039 and *ρ* = 0.664, *P* = 0.007), while IL-1β also positively correlates with birth weight (*ρ* = 0.630, *P* = 0.004). These alterations are in line with increased inflammation associated with macrosomia.

HOMA_IR negatively correlates with NKT cell frequency (*ρ* = −0.645, *P* = 0.029), and positively with TNF-α (*ρ* = 0.571, *P* = 0.041), while maternal age positively correlates with CD4 Naïve T-cells frequency (*ρ* = 0.744, *P* = 0.014), and negatively with IL-18 (*ρ* = 0.654, *P* = 0.002).

### Increased IL-17A in the placenta of GDM non-obese participants

**Figure 2.**
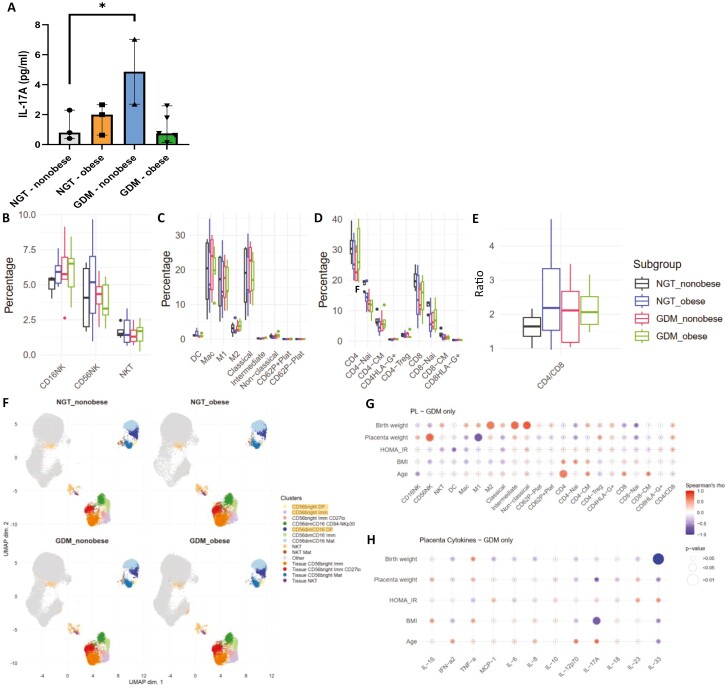
Pro-inflammatory cytokines and immune cell frequencies in placenta. (**A**) IL-17A abundance measured in NGT (non-obese, *n* = 3; obese, *n* = 3) and GDM (non-obese, *n* = 3; obese, *n* = 5) pregnancies. Frequency of NK-cell subsets (**B**), myeloid subsets (**C**), and T-cell subsets (**D**), shown as percentage of live leukocytes are measured in NGT (non-obese, *n* = 4; obese *n* = 6) and GDM participants (non-obese, *n* = 5; obese *n* = 5). (**E**) CD4/CD8 T-cell ratio. (**F**) UMAP was calculated using the full 12-colour panel for NK subsets, altered populations are highlighted in yellow. (**G**) Correlations of clinical parameters with immune cell frequencies and (**H**) cytokine abundance. Statistical significance was calculated using linear regression with maternal age included as a confounder, followed by Benjamini–Hochberg *P*-value correction for multiple testing. Differential analysis of cluster abundance was calculated using R (diffcyt package). The correlation was calculated using Spearman’s rank correlation. Significance levels: *P < 0.05, **P < 0.01, ***P < 0.001.

GDM is a metabolic condition that first presents during pregnancy, and the placenta exerts a causative role in the pathology, in particular, in promoting insulin resistance. Therefore, we measured inflammatory mediators produced by the placenta. Of the 13 cytokines measured, placental production of the pro-inflammatory cytokine IL-17A (*P* = 0.0361) was significantly increased in GDM non-obese compared to NGT non-obese, suggesting a dysregulation that is independent of obesity (Fig. 2A). We equally analysed the immune cell population frequencies and activation marker expression, but found no alterations across all parameters (Fig. 2B–E). Diagnosed treatment (diet-only, metformin, or insulin) had no significant effect on cytokines or immune cell frequencies or activation status in GDM participants.

However, using unsupervised clustering analysis, we identified an NK-cell phenotype related to obesity, with clusters of CD56^bright^ immature NK, CD56^bright^ CD62L^+^KIR2D^+^ (double positive) transitioning NK, and CD56^dim^CD16^+^ double-positive NKs significantly decreased in NGT obese women compared to non-obese, the latter population being also decreased in GDM non-obese (Fig. 2F; Supplementary Fig. S5). To evaluate whether these alterations relate to plasma cytokine levels or immune cell frequencies, we measured their correlation with clinical parameters (Fig. 2G and H). Within the GDM group, placental CD56^bright^ NK frequency is positively correlated with placental weight (*ρ* = 0.714, *P* = 0.047), while M1 macrophages are inversely correlated (*ρ* = −0.714, *P* = 0.047). M2 macrophages, intermediate and non-classical monocytes positively correlate with birth weight (*ρ* = 0.636, *P* = 0.048, *ρ* = 0.697, *P* = 0.025, and *ρ* = 0.758, *P* = 0.011, respectively). Similar to the evidence in maternal circulation, the percentage of CD4 T cells increases with maternal age (*ρ* = 0.633, *P* = 0.049). Placental IL-17A inversely correlates with BMI (*ρ* = −0.714, *P* = 0.047).

These results confirm the presence of subclinical inflammation in the placental microenvironment of GDM, with lower levels of inflammatory NK cells present in NGT obese women who do not develop GDM.

### IFN-γ is increased in VAT of GDM non-obese participants

**Figure 3.**
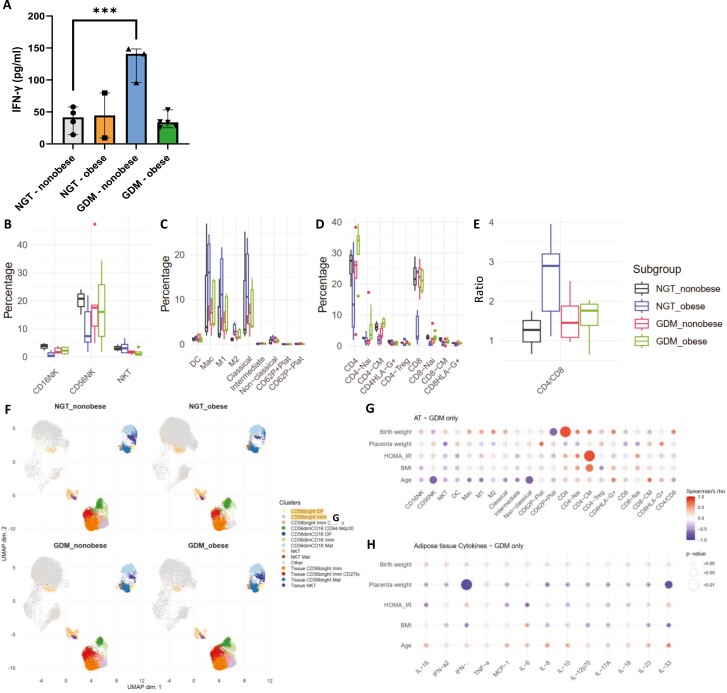
Pro-inflammatory cytokines and immune cell frequencies in visceral adipose. (**A**) IFN-γ abundance measured in NGT (non-obese, *n* = 4; obese, *n* = 3) and GDM (non-obese, *n* = 3; obese, *n* = 5) pregnancies. Frequency of NK-cell subsets (**B**), myeloid subsets (**e**), and T-cell subsets (**D**), shown as percentage of live leukocytes are measured in NGT (non-obese, *n* = 4; obese, *n* = 6) and GDM participants (non-obese, *n* = 5; obese, *n* = 5). (**E**) CD4/CD8 T-cell ratio. (**F**) UMAP was calculated using the full 12-colour panel for NK subsets, altered populations are highlighted in yellow. (**G**) Correlations of clinical parameters with immune cell frequencies and (**H**) cytokine abundance. Statistical significance was calculated using linear regression with maternal age included as a confounder, followed by Benjamini–Hochberg *P*-value correction for multiple testing. Differential analysis of cluster abundance was calculated using R (diffcyt package). The correlation was calculated using Spearman’s rank correlation. Significance levels: *P < 0.05, **P < 0.01, ***P < 0.001.

Obesity is a prominent risk factor for GDM, and AT dysregulation plays a pivotal role in immune-metabolic diseases, including T2D. Therefore, we measured inflammatory cytokines produced by the omental VAT in our study. IFN-γ was significantly increased (*P* < 0.001) in GDM non-obese patients when compared to NGT non-obese (Fig. 3A). There was no significant difference in any of the remaining 12 cytokines measured. We also analysed the immune cell population frequencies and activation marker expression but found no alterations across all parameters (Fig. 3B–E). Diagnosed treatment (diet-only, metformin, or insulin) had no significant effect on cytokines, immune cell frequencies, or activation status in GDM participants. However, with unsupervised clustering and analysis, a mixed NK/T-cell phenotype was identified, namely clusters of CD56^bright^ immature NK and CD56^bright^ double-positive transitioning NK which were significantly decreased in obese NGT patients only, as well as two clusters of CD4 central memory T cells, and various CD8 clusters (Naïve, central memory, and HLA-DR^+^ effector memory; Fig. 3F; Supplementary Fig. S6).

To evaluate whether these alterations relate to plasma cytokine levels or immune cell frequencies, we measured their correlation with clinical parameters in GDM. IFN-γ and IL-33 were inversely correlated with placental weight (*ρ* = −0.857, *P* = 0.007, and *ρ* = −0.809, *P* = 0.015, respectively). CD56^bright^NK and non-classical monocytes frequency also inversely correlate with maternal age (*ρ* = −0.672, *P* = 0.047, *ρ* = −0.685, *P* = 0.029). CD4 T cells were positively correlated with birth weight (*ρ* = 0.8, *P* = 0.009), while central memory CD4 subset correlates with both HOMA_IR (*ρ* = 0.817, *P* = 0.007) and maternal BMI (*ρ* = 0.684, *P* = 0.042). These results suggest that low-level inflammation extends to the AT in GDM non-obese women, and lower immune cells infiltrate characterizes obese women without GDM.

### Alterations in both NK-cell and T-cell phenotypes in NGT obese, GDM obese, and GDM non-obese participants

**Figure 4.**
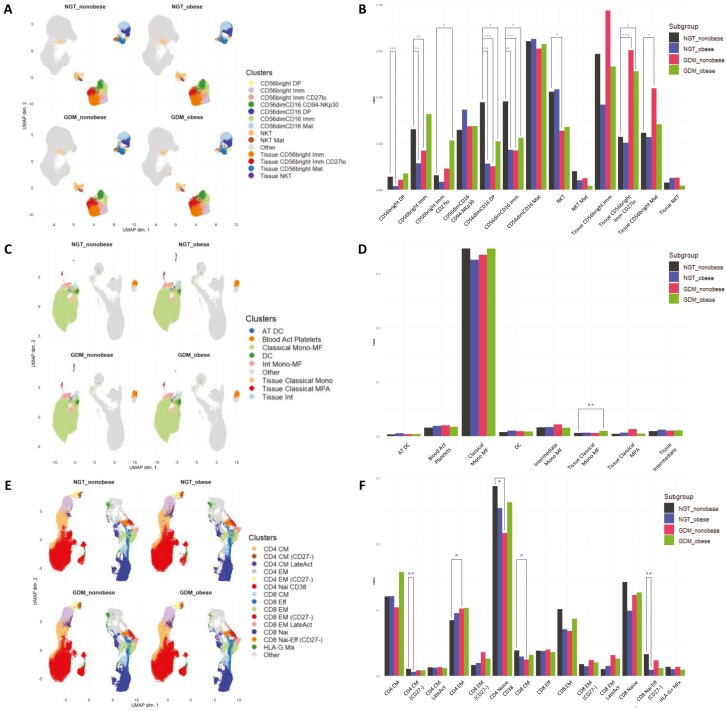
UMAPs were calculated by combining plasma, VAT, and placental tissue to highlight patterns of systemic alterations in (**A, B**) NK subpopulations, (**C, D**) monocytes–macrophages–platelets, and (**E, F**) T-cell populations. (B, D, and F) Barplots indicating the relative abundance of each cluster. Differential analysis of cluster abundance was calculated using R (diffcyt package). Significance levels: *P < 0.05, **P < 0.01, ***P < 0.001.

Given the NK- and T-cell phenotypes identified in independent tissue sites in participants, performed unsupervised clustering and analysis of how all cell markers performed across the three biological sites (maternal circulation, VAT, and placenta) simultaneously was investigated, allowing for the identification of subsets or functional statuses. When combining all sample sites, there were a number of different NK- and T-cell phenotypes that were both unique and shared amongst the participant study groups. Clusters of CD56^dim^ CD16 immature NK and CD56^dim^ CD16 double-positive NK cells were significantly decreased across obese NGT, obese GDM, and GDM non-obese participants compared to non-obese NGT participants. Further, NK-cell clusters, namely CD56^bright^ immature cells, were significantly decreased in both obese NGT and GDM non-obese participants. In NGT obese participants only, CD56^bright^ double-positive NK cells were significantly reduced while CD56^bright^ immature CD27 positive NK cells were significantly increased in GDM obese only compared to non-obese NGT participants. Tissue CD56^bright^ immature CD27 positive NK-cell clusters were unique to GDM regardless of BMI compared to non-obese NGT participants, while an additional tissue CD56^bright^ mature NK-cell cluster was significantly increased in GDM non-obese participants (Fig. 4A and B).

Clusters of T-cell populations were also altered in our study groups, CD4 central memory T cells and CD8 Naïve effector CD27^−^ cells were both significantly decreased in NGT obese participants compared to NGT non-obese participants. In non-obese GDM participants, CD4 effector memory cells were significantly increased, while CD4 Naïve CD38 and CD8 central memory cells were significantly decreased compared to NGT non-obese participants. A natural killer T-cell cluster was significantly decreased in non-obese GDM participants only when compared to NGT non-obese participants (Fig. 4E and F). Finally, a classical tissue monocyte cluster was significantly increased in GDM obese participants only (Fig. 4C and D).

### No significant changes were found in exosomes’ surface marker abundance between study groups

**Figure 5.**
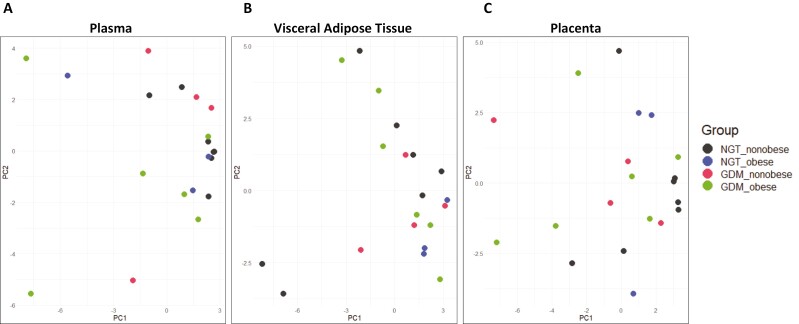
Principal component analysis of EV surface marker abundance in (**A**) maternal plasma, (**B**) VAT, and (**C**) placenta.

Having observed low-level alterations across different tissues and in the circulation, we speculated a systemic communication network of immune-metabolic factors, possibly extending beyond soluble factors and cytokines. EVs are known to play a pivotal role in long-distance communication, carrying information across the body [16]. Therefore, we characterized 37 surface markers on EVs in maternal circulation, VAT explant, and placental tissue explant supernatants, respectively. We used PCA to characterize plasma samples and explant supernatants on the basis of the markers detected. There was a lack of specific differentiation across the different study groups in both maternal plasma and tissue explant supernatants (Fig. 5A–C), which indicated that no single marker, or combination of markers, differentiates between the study groups. Indeed, no significant difference was found in the abundance of any marker in either plasma or tissue explants.

## Discussion

The prevalence of maternal obesity is increasing globally and predisposes women to an increased risk of pregnancy complications including GDM. In this work, we describe the immunological landscape of GDM and its relationship with obesity, at a systemic level. We classified NGT and GDM participants, according to their BMI, into either non-obese (<30 kg/m^2^) or obese (≥30 kg/m^2^) cohorts. We found an increase in both IL-6 and IL-17A in the circulation of obese, GDM participants, while IL-12p70 was significantly increased in non-obese, GDM participants. In the placenta, IL-17A was found to be elevated in GDM non-obese women, and minor NK subsets were decreased in the obese, NGT group. In VAT, IFN-γ was also increased in non-obese GDM women, while NK- and T-cell subsets were reduced in the NGT obese group. When all biological sites were considered together in participant study groups, a number of different NK- and T-cell phenotypes were significantly altered in NGT obese, GDM non-obese, and GDM obese participants, while a cluster of tissue classical monocytes was significantly increased in GDM obese participants only. Finally, there was no significant difference in the abundance of EV surface marker expression in maternal circulation or VAT and placental tissue explants.

In the maternal circulation, GDM obese participants had elevated levels of both IL-17A and IL-6, which is in line with previous findings of increased Th1 and Th17 activity in GDM [21, 22] and its direct correlation with maternal BMI [6]. IL-17 immunity is implicated in both T1D [23, 24] and T2D [25], and IL-6 has been shown to regulate Th17 immunity [26] and play a role in T2D pathogenesis [27]. The increased levels of IL-12p70 in GDM non-obese women suggest an involvement of monocyte/DC immunity that is not directly related to obesity. In fact, IL-12 is increased in T2D non-obese patients [28]. Despite the significant cytokine alterations, there were no significant changes in the frequency of T-cell subsets, or any other immune population in the circulation, or their activation status.

We further investigated potential correlations between immune mediators and clinical parameters and identified that pro-inflammatory monocyte-macrophages positively correlate with birth weight, while IL-1β, IFN-γ, and IL-8 correlated with placental weight. These inflammatory mediators have been previously implicated in macrosomia [29–31]. Non-classical monocytes are often viewed as anti-inflammatory, so inverse correlation with placenta weight is in line with these observations. We also found an inverse correlation of HOMA_IR with NKT cell frequency and a positive correlation with TNF-α. While TNF-α is indicative of inflammation and is commonly associated with IR and GDM [32], there is contrasting evidence on the role of NKT in insulin resistance; however, this is currently based on preclinical evidence in murine models [33] and warrants further exploration in human studies. Interestingly, there was an increase in naïve CD4 T cells and decrease in IL-18 with increasing maternal age, in contrast with recent publications that reported an increase in effector T cells both in mice [34] and humans [35].

Given the critical role of the placenta in terms of contributing to the pathology of GDM, in part due to secretion of both hormones, adipokines and cytokines, we expanded our investigation to assess the placental inflammatory phenotype in GDM. Placental IL-17A was the only cytokine which was significantly increased in the study groups when compared to non-NGT participants, and surprisingly was increased in the GDM non-obese participants only. Placental production of IL-17A is likely due to macrophages [36–38] and contributes to a Th17 inflammatory phenotype. However, there was no difference in frequency or activation of macrophages, T cells, and other immune cells populations in the placenta.

NK cells have been found to be reduced in placenta of obese women [39] with altered function [40], in concordance with our results. To date, only one study reported that NK cells are increased in the placenta of GDM pregnancy [41], indicating possibly a rarer phenotype that is masked in our cohort. NK cells are important in the early phases of pregnancy, contributing to tissue remodelling and embryo implantation. However, little is known about their role in later stages and at term, when their number are normally greatly reduced. Upon investigation of potential correlations between placental inflammatory mediators and clinical parameters, anti-inflammatory monocyte-macrophages (M2, intermediate, and non-classical) correlated with increased birth weight. Previously, M2 macrophages have been shown to be increased in the decidua parietalis of obese women with uncomplicated pregnancy [42]. Similar to maternal circulation, CD4^+^ T cells and inflammatory cytokines IL-12p70 and IL-17A are increased with maternal age, suggesting increased inflammation with advanced maternal age, a common risk factor for many pregnancy complications.

AT inflammation is a common feature of T2D, where macrophages infiltrate this metabolic tissue with the resultant production of pro-inflammatory cytokines triggering insulin resistance [43, 44]. Increased IFN-γ in AT of non-obese GDM women reflects a Th1 inflammatory phenotype that is common in dysfunctional AT [45]. IFN-γ has been found to be produced by intestinal T-helper in T2D non-obese patients [46], suggesting that the phenotype we observe is a typical feature of diabetes and to a lesser extent of obesity. Indeed, it has been shown that IFN-γ interferes with insulin signalling, glucose uptake, and lipid storage in human adipocytes, contributing to IR but not increased adiposity [47]. IFNγ-producing NK cells in AT are associated with hyperglycaemia and insulin resistance in obese women [48], while a deficiency of NK cells or IFN-γ was shown to prevent the accumulation of pro-inflammatory macrophages in VAT and greatly ameliorate insulin sensitivity [49]. Therefore, the reduced frequency of NK and T cells we observe in NGT obese women could be protecting from the development of GDM. When investigating correlations between immune mediators in VAT and clinical parameters in the whole GDM group, we found that IFN-γ and IL-33 were inversely correlated with BMI and placental weight. IL-33 production in AT is associated with IR and T2D [50], while memory T cells have previously been reported to correlate with HOMA_IR and BMI [51].

When unsupervised clustering of all immune populations from maternal circulation, VAT and placenta combined in all study groups was performed, additional modifications in NK- and T-cell subsets were evident in NGT obese and in both GDM study groups, respectively, when compared to non-obese NGT participants. Clusters of CD56^dim^ CD16 immature NK and CD56^dim^ CD16 double-positive NK cells were decreased in NGT obese and in both GDM study while alterations in additional NK-cell subtypes were unique to specific study groups. One particular cluster of tissue CD56^bright^ immature CD27 positive NK-cell clusters was unique to GDM regardless of BMI. However, it is evident that NK-cell subsets are mainly reduced in pregnancies impacted by either obesity and/or hyperglycaemia. While there are conflicting reports on NK-cell populations in GDM, in the general population, obesity is known to decrease the CD56^dim^ CD16 NK-cell effector subset [52], while this subset have also been shown to be depleted in hyperglycaemia as evident in pregnant women with T1D [53]. The increase in GDM only of tissue CD56^bright^ immature CD27 positive NK-cell clusters which have low cytotoxic capacity but increased levels of cytokine release is in keeping with the elevation in pro-inflammatory cytokines in our GDM participants.

Different subsets of CD4 and CD8 T cells were also reduced in either NGT obese or non-obese GDM participants, but surprisingly there was no evidence of alteration in any T-cell subset in obese GDM participants. The reduction in CD4 central memory cells in NGT obese participants may be protective against the development of diabetes as this specific population is increased in T2D [54]. A reduction in NKT cells was only identified in non-obese GDM participants, while CD4 effector memory cells were the only T-cell population increased across study groups and this increase was also specific to non-obese GDM participants. While we did not discriminate between NKT cell subgroups in this study, their reduction in non-obese GDM participants may indicate a non-classical NKT subgroup as this subset promotes insulin sensitivity [9]. The cluster of CD4 effector memory T cells which were increased in non-obese GDM participants only have previously been identified in T1D pathogenesis where they may support B-cell production of insulin autoantibodies [55]. Conversely, a cluster of tissue classical monocytes was significantly increased in GDM obese participants only. The increase in this cluster is indicative of the increase in M1 macrophage populations typically described in T2D associated with obesity [56]. This increase has also been reported in placental inflammation in pregnant women with obesity [57], additionally, we have shown that elevated production of mitochondrial superoxide by placental macrophage populations contributes to increased inflammation in GDM [58].

EVs are important mediators of physiological functions by acting as signalling messengers across organs and are important in the maternal–foetal communication and regulation of maternal metabolism [16]. It has previously been shown that injection of EVs from GDM pregnancies can induce GDM in otherwise healthy pregnant mice [18]. However, in our study, we do not observe significant differences in EVs surface markers, across maternal circulation or in either VAT or placental tissue explant supernatants, respectively, suggesting that the exosome communication network is not significantly perturbed. Markers on the EV surface can also indicate the cell of origin, pointing to specific contributions in case of alterations. We do not observe any such contribution. However, this does not exclude the possibility that the content of EVs is significantly altered. Further functional studies are required to clarify EVs contribution to human GDM pathology.

In this study, strict exclusion criteria ensured that only nulliparous women, without significant comorbidities or physiological adaptive changes from previous pregnancies, were included. Women were recruited on the day of caesarean section, with identical methods of sample collection and processing used for each participant. However, some limitations do exist. Firstly, our study is predominantly cross-sectional in nature, eliminating the possibility to derive causation from our findings. This research was carried out in a largely White participant group, thereby limiting the extrapolation of our findings to other ethnicities. As our observations are conducted at term, GDM has been managed for several months either by diet alone or with pharmacological intervention, which may have impacted inflammation. Another limitation of our study is the small sample number in subgroups. A larger cohort would allow for better stratification of participants and reduce the effects of confounders such as maternal age. Nonetheless, this study is the first of its kind to perform an in-depth identification of a broad panel of immune cells, inflammatory and EV markers across multiple tissues in the same participants with GDM.

Metainflammation is defined as a modest, low-grade response that is chronically maintained by metabolic cells, potentially driven by several factors in the local tissue environment, impacting cell metabolism and therefore cytokine production. We speculate that the inflammatory phenotype identified in this study is indicative of metainflammation, as only minor cytokine alterations are evident in GDM, while immune cell frequencies and activation status were largely unchanged across various tissues, even in GDM obese participants. While there is wide agreement that increased adiposity assimilates with increased inflammation in the non-pregnant state, this overt relationship may not be as evident during pregnancy and warrants further examination in future longitudinal studies.

## Supplementary Material

uxae010_suppl_Supplementary_Figures_S1-S6_Table_S1

## Data Availability

The datasets generated and analysed, and resources used, for the current study are available from the corresponding author upon reasonable request.

## Abbreviations

AT: adipose tissue
BMI: body mass index
EDTA: ethylenediaminetetraacetic acid
EVs: extracellular vesicles
GDM: gestational diabetes mellitus
MCP-1: monocyte chemoattractant protein 1
NGT: normal glucose tolerant;
VAT: visceral adipose tissue
