# Supplementary material for: Exposure to gestational diabetes mellitus increases subclinical inflammation mediated in part by obesity
Source: Clin Exp Immunol. 2024 Feb 9;216(3):280–92. doi: 10.1093/cei/uxae010 (PMC11097910; doi:10.1093/cei/uxae010)
Supplement: uxae010_suppl_Supplementary_Figures_S1-S6_Table_S1 [file uxae010_suppl_supplementary_figures_s1-s6_table_s1.pdf]

Antibody	Clone	Fluorochrome	Manufacturer	Cat. No
**Panel 1** - NK cells				
CD94	REA113	FITC	Miltenyi	130-123-678
NKp44	P44-8	PerCP-Cy5.5	BioLegend	325114
KIR2D	REA1042	PE	Miltenyi	130-117-478
CD27	M-T271	PE-Dazzle594	BioLegend	356422
CD40L	24-31	PE-Cy5	BioLegend	310808
NKp30	P30-15	PE-Cy7	BioLegend	325214
CD56	MEM188	AlexaFluor647	BioLegend	304612
CD16	3G8	AlexaFluor700	BioLegend	302026
NKG2D	1D11	APC-Cy7	BioLegend	320824
NKp46	9E2	eFluor450	eBioscience	48-3359-42
CD3	UCHT1	eFluor506	eBioscience	69-0038-42
CD62L	DREG-56	BV605	BioLegend	304834
**Panel 2** - Monocytes/Macrophages/Platelets				
CD62P	AK-4	FITC	eBioscience	11-0628-42
CD64	10.1	PerCP-Cy5.5	BioLegend	305024
CD11c	BU15	PE	BioLegend	337224
HLA-DR	L243	PE-Dazzle594	BioLegend	307654
CD42b	HIP1	PE-Cy5	BD	551141
CD14	61D3	PE-Cy7	eBioscience	25-0149-42
CD206	15-2	AlexaFluor647	BioLegend	321116
CD16	3G8	AlexaFluor700	BioLegend	302026
CCR2	K036C2	APC-Cy7	BioLegend	357220
DC-SIGN	REA617	VioBlue	Miltenyi	130-110-450
CD11b	ICRF44	eFluor506	eBioscience	69-0118-42
CD86	BU63	BV605	BioLegend	374214
**Panel 3** - T cells				
CD4	OKT4	AlexaFluor488	eBioscience	53-0048-42
CD8a	RPA-T8	PerCP-Cy5.5	BioLegend	301032
HLA-G	87G	PE	Miltenyi	130-099-843
HLA-DR	L243	PE-Dazzle594	BioLegend	307654
CD45RO	UCHL1	PE-Cy5	BioLegend	304208
CD27	M-T271	PE-Cy7	BioLegend	356412
CD25	BC96	AlexaFluor647	BioLegend	302618
CD127	A019D5	AlexaFluor700	BioLegend	351344
CD45RA	HI100	APC-Cy7	BioLegend	304128
CD38	HIT2	eFluor450	eBioscience	48-0389-42
CD3	UCHT1	eFluor506	eBioscience	69-0038-42
CD62L	DREG-56	BV605	BioLegend	304834

Table ST1 Flow Cytometry panels

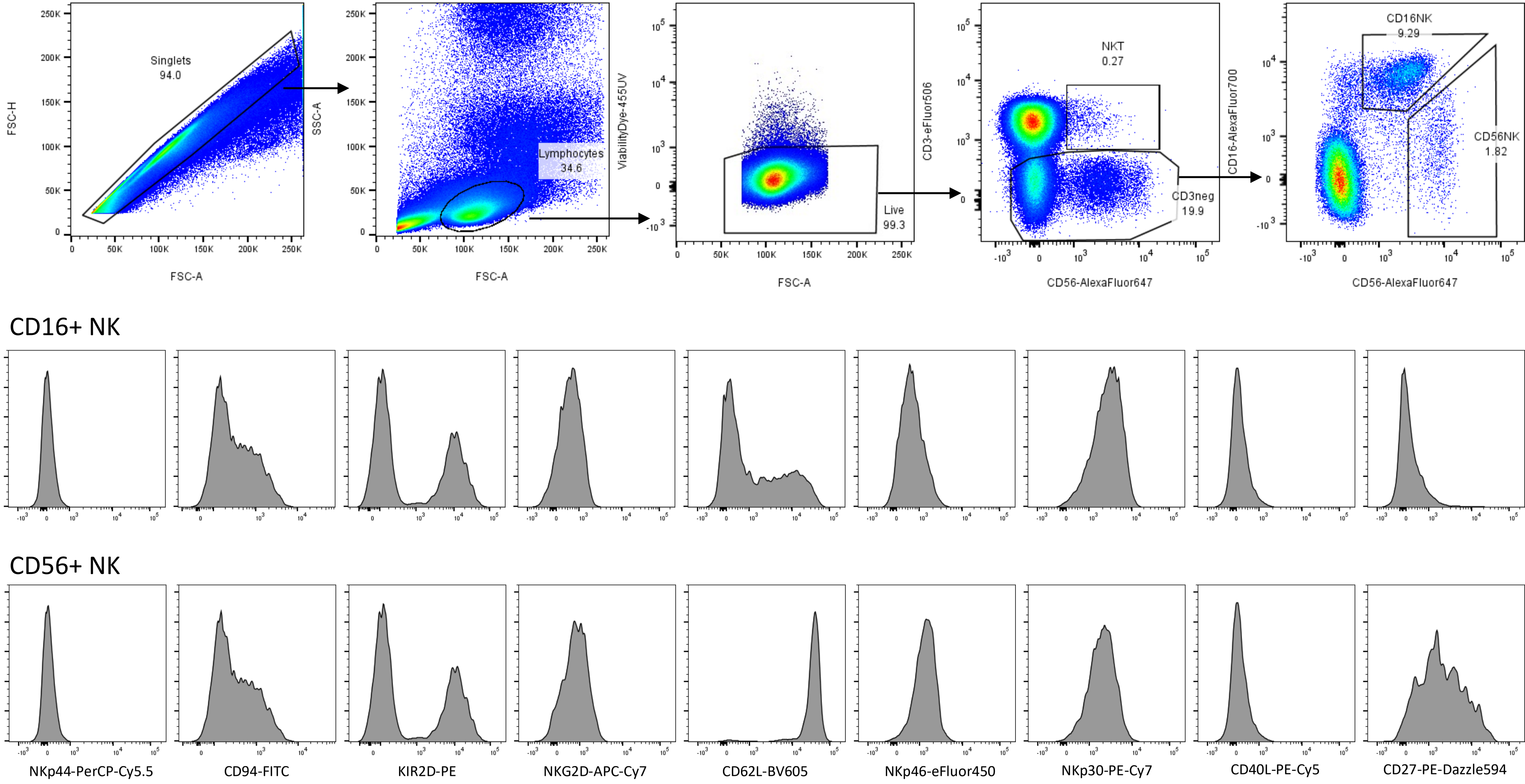

Figure S1. Example gating strategy for panel 1 (NK subsets). Doublets were excluded first, followed by size selection of Lymphocytes. Dead cells were excluded using fixable Viability Dye. NKT cells were gated as CD56+CD3+ cells, and CD3- cells were further gated into CD56+CD16- NK vs CD56lowCD16+ NK.

Activation and maturation markers were measured in each subpopulation.

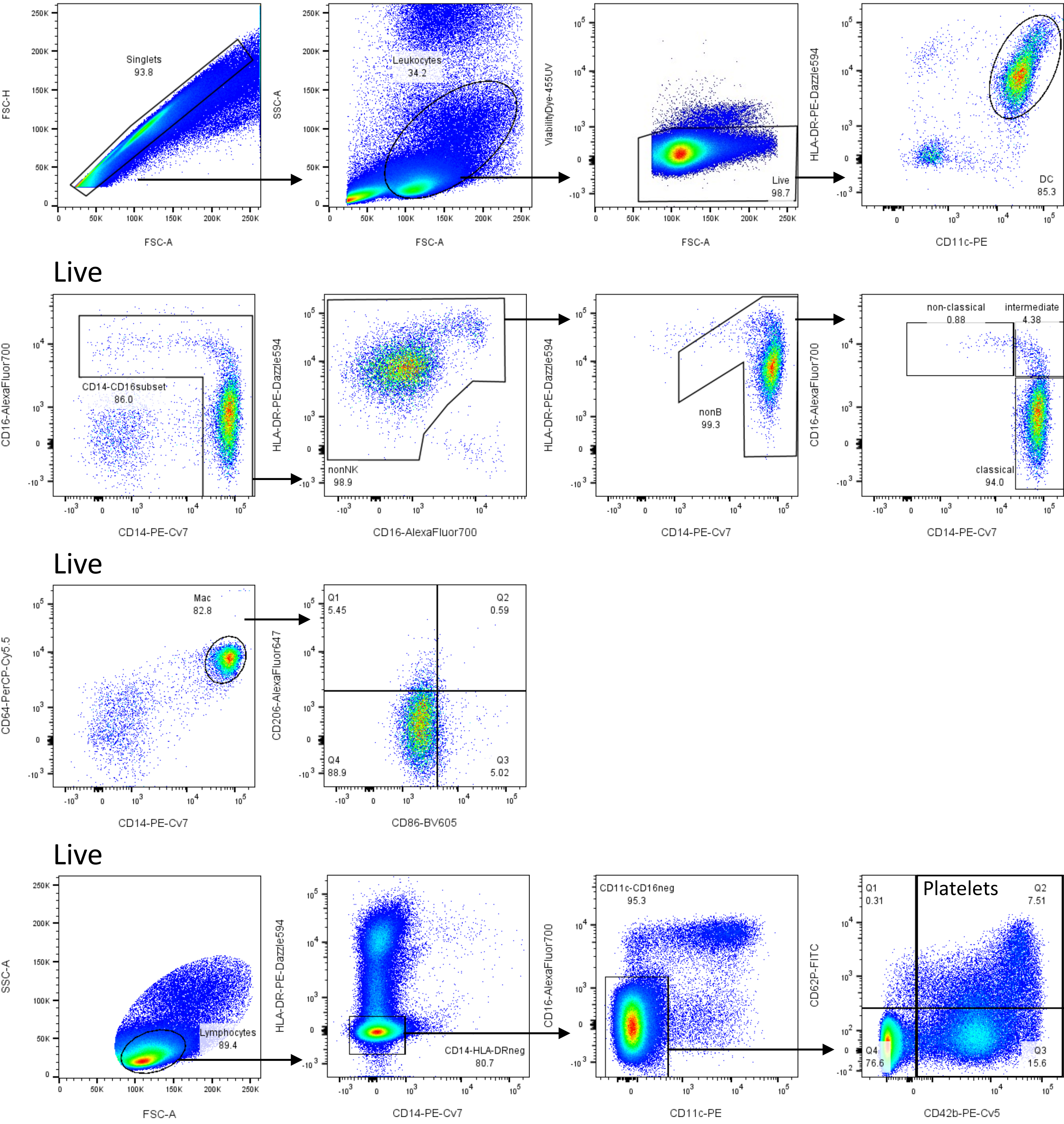

Figure S2. Example gating strategy for panel 2 (Myeloid and platelets subsets). Doublets were excluded first, followed by size selection of leukocytes. Dead cells were excluded using fixable Viability Dye. Dendritic cells were gated as CD11c+HLA-DR+. To gate monocytes, CD16-CD14- cells were excluded, followed by exclusion of NK cells (HLA-DR-CD16high) and B cells (CD14-HLA-DRhigh). Monocyte subsets were identified based on CD14/CD16 expression.

Macrophages were gated as CD14+CD64+, and M1 and M2 subsets distinguished based on CD86/CD206 expression.

Platelets were gated by selecting lymphocytes by size, followed by exclusion of CD14+, HLA-DR+, CD11c+ and CD16+ cells and selection for CD42b expression.

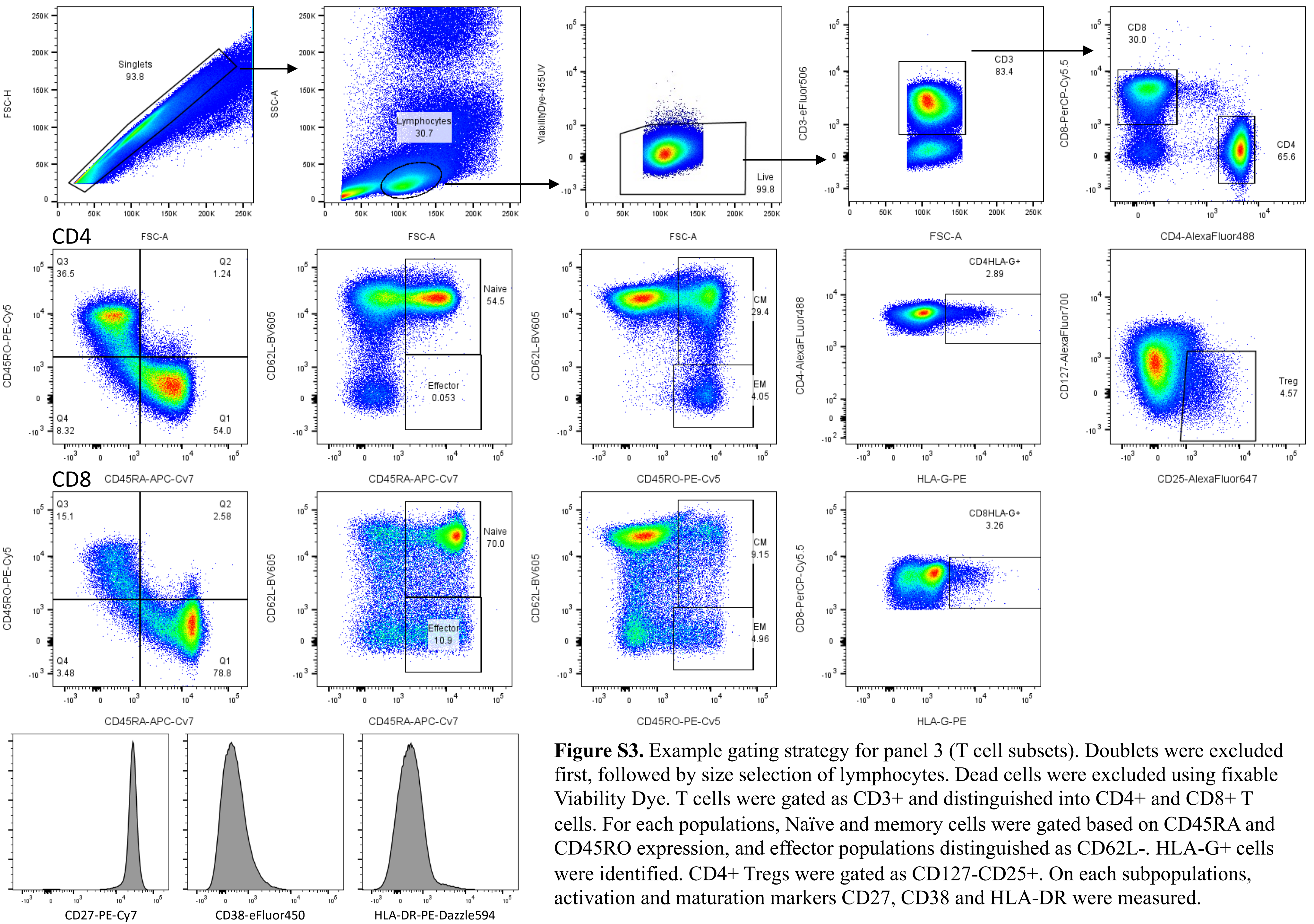

Figure S3. Example gating strategy for panel 3 (T cell subsets). Doublets were excluded first, followed by size selection of lymphocytes. Dead cells were excluded using fixable Viability Dye. T cells were gated as CD3+ and distinguished into CD4+ and CD8+ T cells. For each populations, Naïve and memory cells were gated based on CD45RA and CD45RO expression, and effector populations distinguished as CD62L-. HLA-G+ cells were identified. CD4+ Tregs were gated as CD127-CD25+. On each subpopulations, activation and maturation markers CD27, CD38 and HLA-DR were measured.

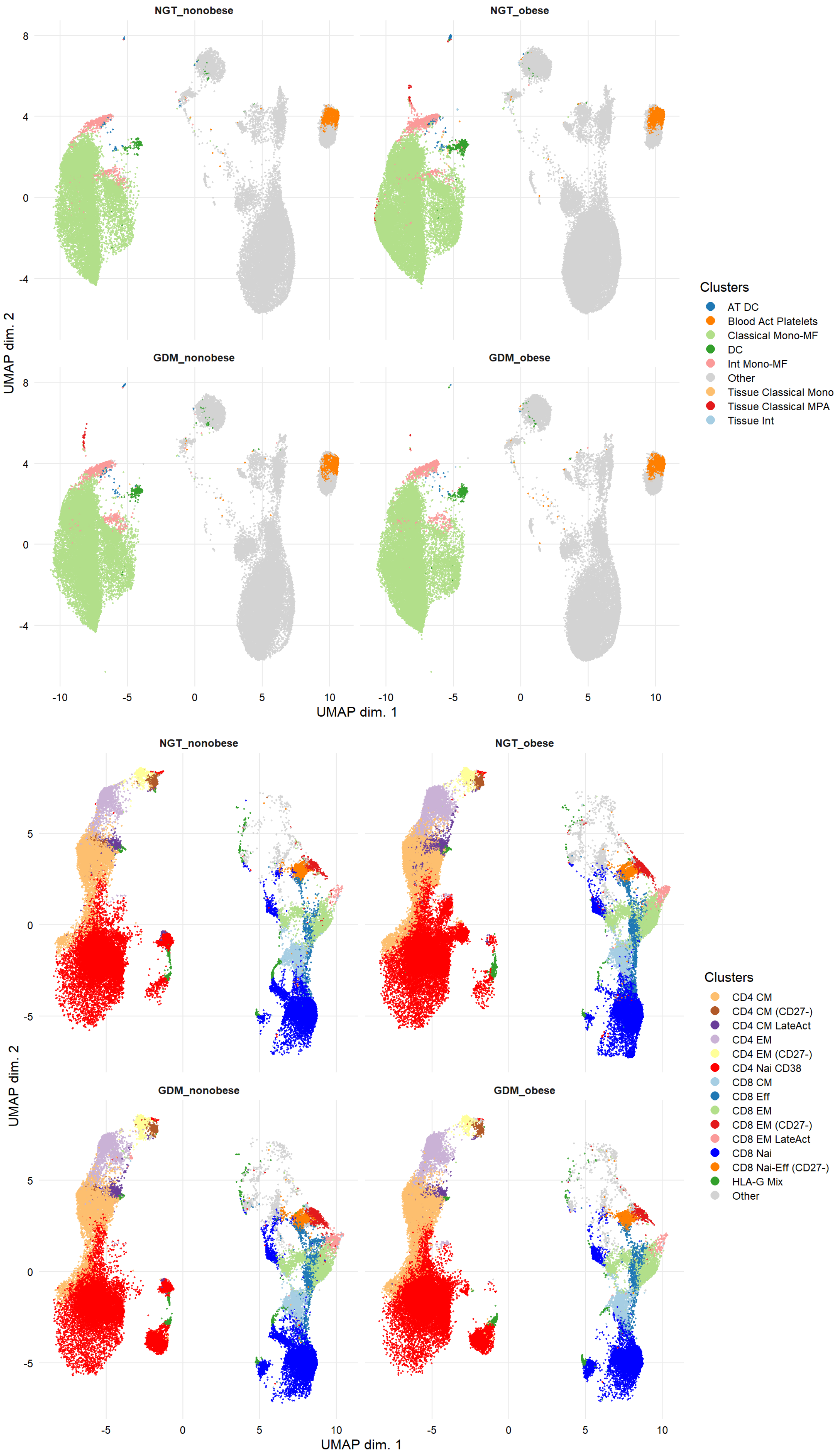

Figure S4 UMAPs of maternal blood, panels 2 (monocytes/macrophages), and 3 (T cells)

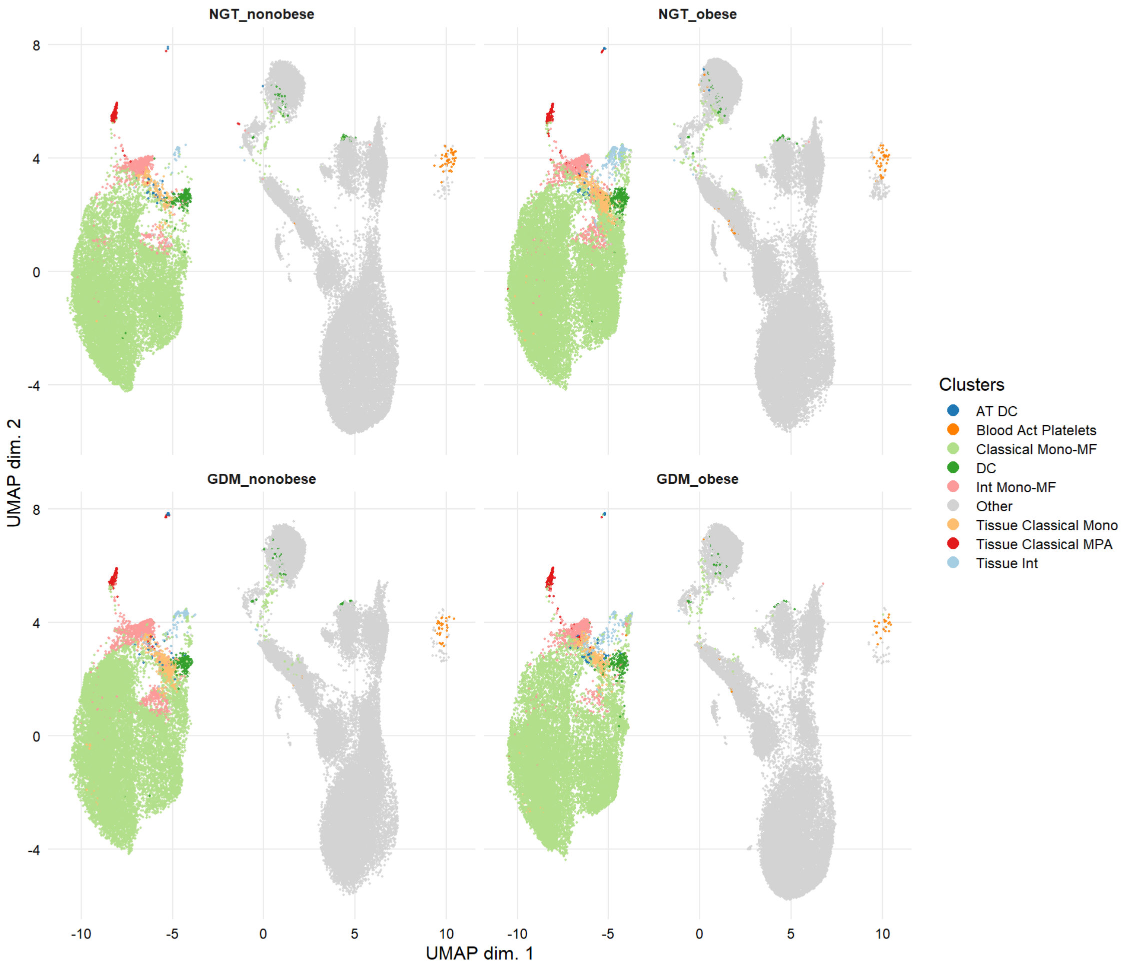

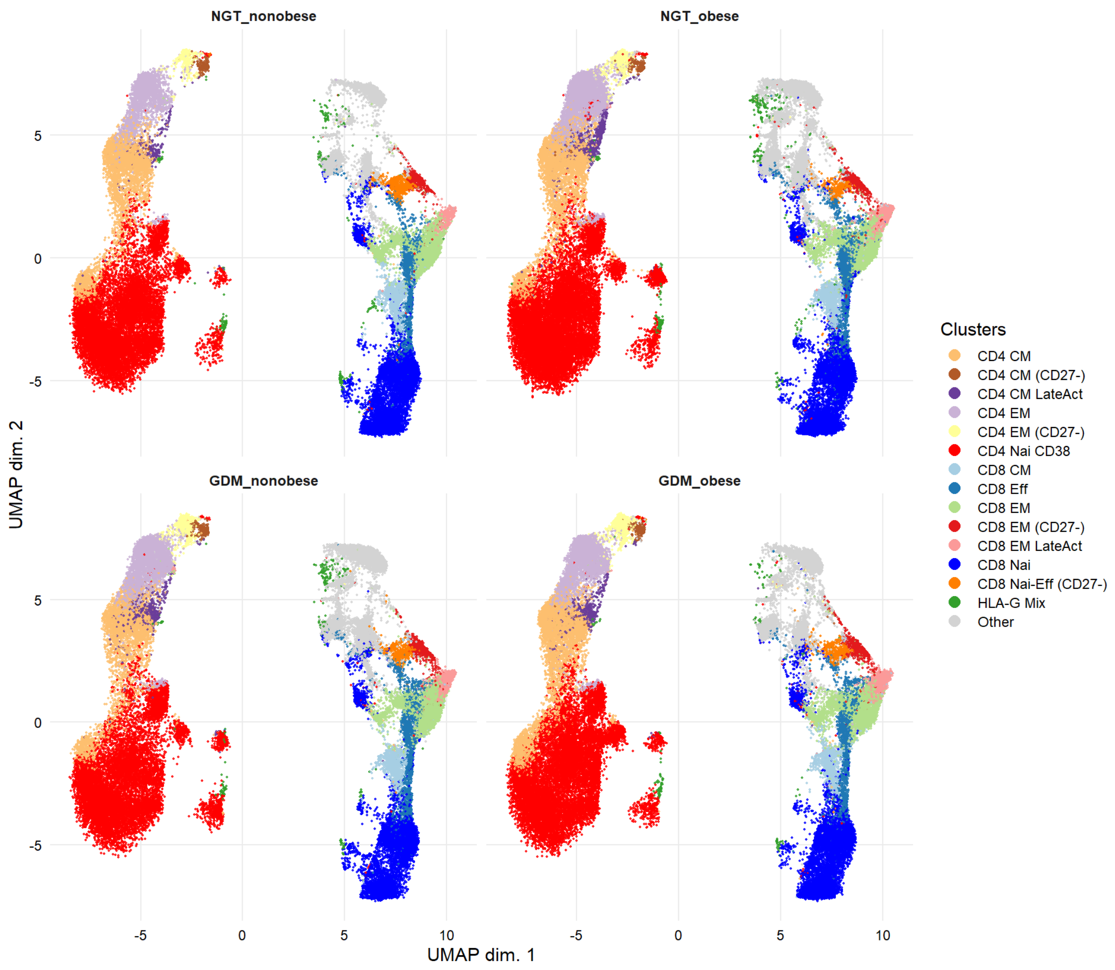

Figure S5 UMAPs of placenta, panels 2 (monocytes/macrophages), and 3 (T cells)

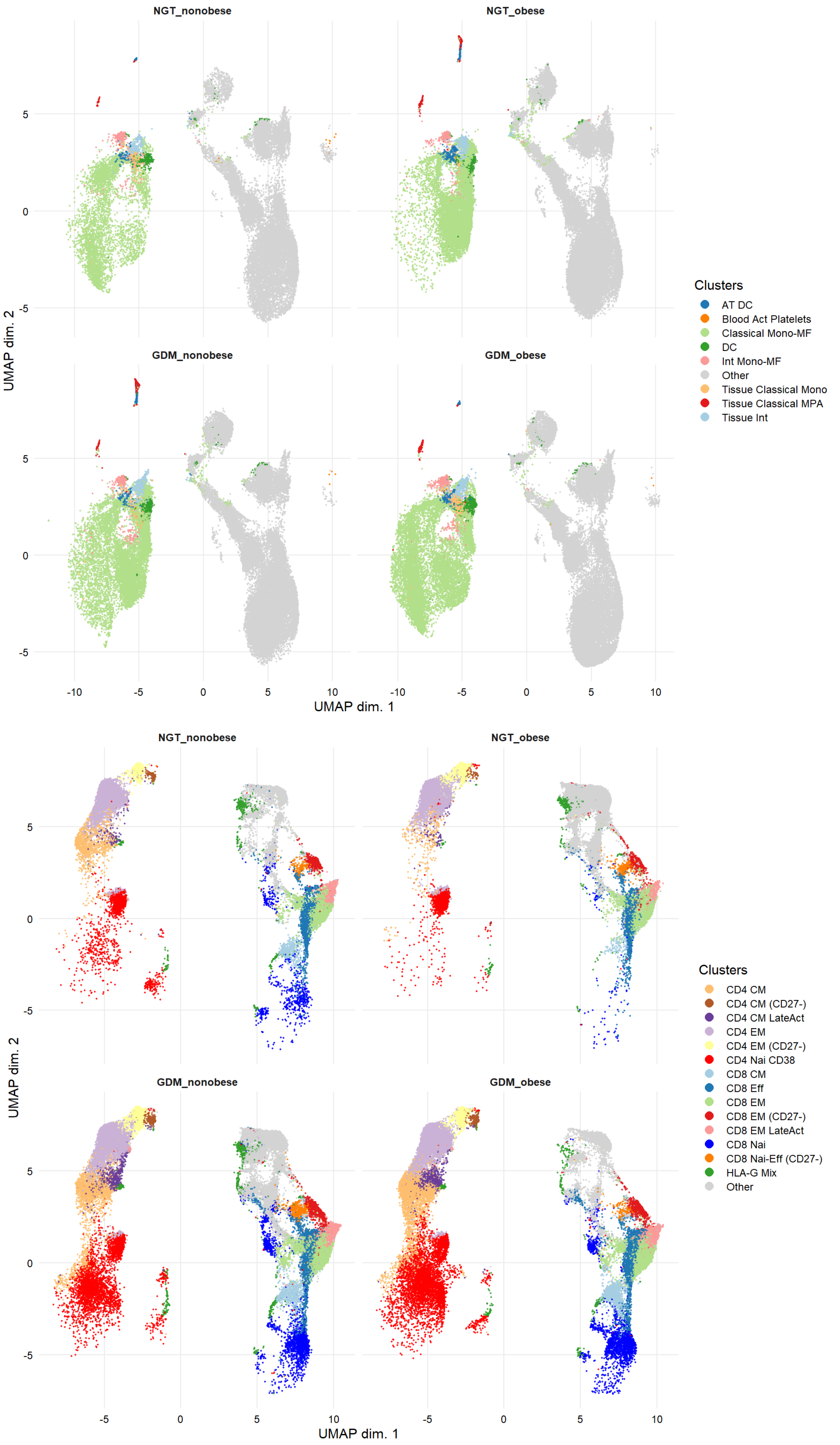

Figure S6 UMAPs of adipose tissue, panels 2 (monocytes/macrophages), and 3 (T cells)
